# Nasal Microbiota, Olfactory Health, Neurological Disorders and Aging—A Review

**DOI:** 10.3390/microorganisms10071405

**Published:** 2022-07-12

**Authors:** Subramanian Thangaleela, Bhagavathi Sundaram Sivamaruthi, Periyanaina Kesika, Muruganantham Bharathi, Chaiyavat Chaiyasut

**Affiliations:** 1 Innovation Center for Holistic Health, Nutraceuticals, and Cosmeceuticals, Faculty of Pharmacy, Chiang Mai University, Chiang Mai 50200, Thailand; thangaleela.s@cmu.ac.th (S.T.); kesika.p@cmu.ac.th (P.K.); bharathi.m@cmu.ac.th (M.B.); 2 Office of Research Administration, Chiang Mai University, Chiang Mai 50200, Thailand

**Keywords:** nasal microbiota, Parkinson’s disease, Alzheimer’s disease, multiple sclerosis, SARS-CoV-2, COVID-19

## Abstract

The nasal region is one of the distinct environments for the survival of various microbiota. The human microbial niche begins to inhabit the human body right from birth, and the microbiota survive as commensals or opportunistic pathogens throughout the life of humans in their bodies in various habitats. These microbial communities help to maintain a healthy microenvironment by preventing the attack of pathogens and being involved in immune regulation. Any dysbiosis of microbiota residing in the mucosal surfaces, such as the nasal passages, guts, and genital regions, causes immune modulation and severe infections. The coexistence of microorganisms in the mucosal layers of respiratory passage, resulting in infections due to their co-abundance and interactions, and the background molecular mechanisms responsible for such interactions, need to be considered for investigation. Additional clinical evaluations can explain the interactions among the nasal microbiota, nasal dysbiosis and neurodegenerative diseases (NDs). The respiratory airways usually act as a substratum place for the microbes and can act as the base for respiratory tract infections. The microbial metabolites and the microbes can cross the blood–brain barrier and may cause NDs, such as Parkinson’s disease (PD), Alzheimer’s disease (AD), and multiple sclerosis (MS). The scientific investigations on the potential role of the nasal microbiota in olfactory functions and the relationship between their dysfunction and neurological diseases are limited. Recently, the consequences of the severe acute respiratory syndrome coronavirus (SARS-CoV-2) in patients with neurological diseases are under exploration. The crosstalk between the gut and the nasal microbiota is highly influential, because their mucosal regions are the prominent microbial niche and are connected to the olfaction, immune regulation, and homeostasis of the central nervous system. Diet is one of the major factors, which strongly influences the mucosal membranes of the airways, gut, and lung. Unhealthy diet practices cause dysbiosis in gut microbiota and the mucosal barrier. The current review summarizes the interrelationship between the nasal microbiota dysbiosis, resulting olfactory dysfunctions, and the progression of NDs during aging and the involvement of coronavirus disease 2019 in provoking the NDs.

## 1. Introduction

All humans and other animals are hosts for many unicellular and multicellular microbial communities, such as bacteria, viruses, fungi, and parasites [1]. Microbes are omnipresent in the environment and exist in the human body. They can adapt or adjust themselves as the host or the environment changes. The microbes and their metabolites might be involved in the host’s health and diseases [2]. The microbes can reside on every surface of the human body [3], such as the oropharynx, nasopharynx, respiratory system [4], digestive tract [5], urinary system, genital organs [6], and the skin [7].

Microbial dysbiosis is the alteration in healthy microbiota composition, which causes pathological conditions leading to health issues [8]. The microbiota can be both transient and resident; their diversity is affected by various factors, such as drugs, surrounding environmental microorganisms [9], habitat, nutritional availability, and host factors, such as host hygiene, immunity, and genetics [10], and physical factors, such as oxygen, pH, moisture, and other microbial interactions [11]. The colonization of opportunistic pathogens results in the onset of respiratory infections and changes in the innate immune mediators [12]. The human respiratory passages start from the opening of the nostrils (nose or anterior nares). They lead to the nasopharynx posteriorly and the lung alveoli [13]. The upper respiratory tract (URT) has a constant airflow. It has the highest density of microbial communities, which prevent the localization and spreading of pathogens on the lower respiratory tract’s (LRT) mucosal surface [14].

The nasal microbiota are different from the microbial community in the URT and remain constant throughout adulthood [15]. Changes in the nasal microbiota may initiate in the middle age of adults. At the age of 40–65 years, the nasal microbiota of healthy adults are completely dominated by *Staphylococcus*, *Streptococcus*, *Veillonella*, *Cutibacterium* and *Corynebacterium* species [16]. The composition of the nasal and oropharyngeal microbiota changes during aging and becomes similar to that of the microbial community of the oropharyngeal region [16,17]. Roghmann et al. examined the diversity of the nasal microbiota among elderly subjects who resided in nursing homes and independent homes. The study revealed that the elderly individuals showed an abundance of *Streptococcus* and a relative abundance of other species, such as *Lactobacillus reuteri*, *Staphylococcus epidermidis*, and *Rothia mucilaginosa*, in their nasal passages [18].

The older adults (≥65 years) with respiratory tract infections (RTI) have Corynebacterium, Moraxella, Staphylococcus, Dolosigranulum, Streptococcus, Haemophilus, Peptoniphilus, Cutibacterium, Anaerococcus, Enterobacteriaceae, Pseudomonas, and Neisseria in the nasal passages. Prevotella, Veillonella, Streptococcus, Neisseria, Fusobacterium, Leptotrichia, Haemophilus, Rothia, Porphyromonas, Actinobacillus, Lactobacillus, and Staphylococcus were found in the oropharynx. Compared to the healthy older adults, Moraxella catarrhalis and M. nonliquefaciens were less prevalent in the elderly individuals with lower respiratory tract infections (LRTI), which indicates the association of *Moraxella* spp. in the respiratory health of the elderly [19]. In contrast, *M. catarrhalis* and *M. nonliquefaciens* were reported to cause RTIs in young children [20].

Some evidence suggests that a few bacteria have a major role in linking the nasal cavity and the central nervous system (CNS). *Chlamydia pneumonia* is an obligate intracellular pathogen responsible for sinusitis and pneumonia and found in the brains of AD patients [21]. The post-mortem studies of AD brains showed an increased load of *Propionibacterium acnes* belonging to the oral, nasopharyngeal, and skin niches [22]. The diphtheria toxin produced by *Corynebacterium diphtheria* can enter the CNS and result in sporadic AD [23]. In addition to neuropathological changes, some changes were found in the mucosal sensory nerve terminals of the oropharynx, larynx, upper esophagus [24], and gut [25] in Parkinson’s disease (PD). In the same way as the involvement of the nasal microbiota in neurodegenerative diseases, the gut microbiota link the gut and brain by inducing bidirectional communication through the integration of the gut–brain immunological mediators [26]. PD is mainly characterized by the loss of dopaminergic neurons in the substantia nigra, due to the accumulation of α-synuclein, otherwise known as Lewy bodies, in the central nervous system [27]. In the case of AD, the neuropathological features include the formation of amyloid-β (Aβ; a short peptide found in the amyloid plaques of the AD brain) plaques and the neurofibrillary tangles of the phosphorylated tau proteins, which result in the loss of neurons and synaptic elements [28,29].

The macronutrients and micronutrients of the diet significantly affect the mucosal barrier, gut, lung, and microbiota [30]. A healthy diet helps enhance the gut–brain axis and is possibly involved in preventing mental disorders [31]. Diet and lifestyle have been associated with neurodevelopmental disorders [32]. The dietary fibers are utilized by the members of the gut microbiota (*Firmicutes*, *Bacteroidetes*, *Bifidobacterium* and *Prevotella*), resulting in the production of short-chain fatty acids (SCFAs) [33], such as acetate, propionate, and butyrate, which are crucial for gut epithelial and immune regulations [34], the intestinal homeostasis, blood–brain barrier (BBB), and the neuroimmunoendocrine functions [35].

The entry site of pathogens and the host’s response affect the disease outcomes. The URT and the nasal barrier play the main role in preventing infection [36]. Even though the nasal mucosa of humans gives rise to varied microbial communities, in the case of the coronavirus disease 2019 (COVID-19) pathogenicity, the URT was acting as the main site of entry [36]. The COVID-19 pathogenesis may be linked to the nasal or respiratory tract microbiota [37]. Yet the role of the microbiota in the upper airways in severe acute respiratory syndrome coronavirus-2 (SARS-CoV-2) infection needs to be studied in detail. The current review summarizes the interrelationship between the nasal microbiota dysbiosis, respiratory tract infections, olfactory dysfunctions, and the progression of NDs during aging. The manuscript also highlights the diet–microbiota–brain interrelationship and the involvement of COVID-2019 in provoking the NDs.

## 2. Nasal Microbiota and Respiratory Tract Infections

The colonization of the microbes inside the human body is initiated immediately after birth [38]. The first microbial colonization in the nasopharyngeal region of infants resembles the maternal vaginal or skin microbiome [39]. The initial microbial exposure will determine the further successive microbial entries and lead to stable ecosystems during adulthood [40]. With an increase in age, the nasal microbiota are dominated by species of *Moraxella*. On the contrary, the abundance of *Haemophilus* and *Streptococcus* species leads to less stable microbiota [41]. The LRT microbiota of premature infants are dominated by pathogenic *Staphylococcus* spp. [42], *Ureaplasma* spp. [43], and *Acinetobacter* spp. [44]. The microbiota of the URT of infants differs from adults. The nasal microbiome of children is highly dense, with a less diverse population [17,45]. The anterior nares of adults are rich in Actinobacteria and Firmicutes, and less dominated by Bacteroidetes [46,47,48]. Healthy individuals (18 to 65 years old) are rich in *Staphylococcus* spp., *Corynebacterium* spp., *Dolosigranulum* spp., *Moraxella* spp., *Streptococcus* spp., or *Fusobacterium* spp. in their URT. Specifically, *Streptococcus* spp. and *Fusobacterium* spp. are colonized in the nasopharynx and completely absent in the nose [49]. The nasal passages of humans are inhabited by commensal pathobionts, such as *Staphylococcus aureus*, *Haemophilus influenzae*, *Streptococcus pneumoniae*, and *Moraxella catarrhalis* [50]. In older adults (68-96 years old), the nostrils were dominated by the non-pneumococcal *Streptococcus* [16]. The initial acquisition of microbes and their establishment is a complex multistage process. The microbiota of the nasopharynx, oropharynx, and lungs play an important role in the immune system, metabolism, neuro-regulation, and several respiratory diseases, such as upper respiratory tract infections (URTIs) [51].

The microbial niches in the URT and LRT are different. They are influenced by various factors, such as mode of birth, feeding patterns, lifestyle, immunity, and vaccinations [52,53]. The URTIs include the common cold, laryngitis, pharyngitis/tonsillitis, allergic rhinitis, acute rhinitis, acute rhinosinusitis, and otitis media. Lower respiratory tract infections (LRTIs) include acute bronchitis, bronchiolitis, pneumonia, and tracheitis [54]. The pathogenic microbes colonizing the URT can be relocated into the LRT and lung, causing respiratory diseases [55,56]. Recent techniques, such as next-generation sequencing, show a diverse range of microbial species in the LRT (Table 1). The ambient air and the gastric–esophageal reflux are responsible for the LRT microbial load [57]. Whelan et al. [16] revealed that the discrete microbiota of the nasal and oropharyngeal region are lost during aging and are replaced with an oropharyngeal-like microbial population enriched with *Streptococcus* spp. It appears that the increase in *Streptococci* spp., such as *S. pneumoniae*, causes pneumococcal infections. The different microbial communities that commonly reside in the URT and LRT are summarized in Table 1.

As nasal microbiota are associated with regulating the immune functions, the dysbiosis of nasal microbiota may be responsible for nasal inflammatory diseases [68]. The inflammation in the sinus and nasal mucosal layers is linked with chronic rhinosinusitis (CRS), one of the common inflammatory diseases of the URT, with the symptoms such as congested nose, sinus pain, headaches, and attention difficulties and depression [69,70,71]. The dysbiosis, damaged immune barrier, inflamed mucosal epithelium, and secondary bacterial overgrowth cause a chronic immune response and inflammation, which may trigger CRS [72].

## 3. Nasal Microbiota and Olfactory Health

The nasal cavity is comprised of different microbes [73]. The respiratory tract is lined with ciliated epithelial cells. The epithelial layer of the nasal cavity acts as a barrier that detects, filters, and helps remove the inhaled microorganisms and dust or unwanted particles [74], and prevents the host system from being infected or the pathogens from reaching the LRT [75]. It clears the airways and the lungs, and the mucus secreted by the interspersed goblet cells hydrates the airways [74,76]. The respiratory mucosa and the motile cilia in the respiratory tract play a major role in protecting the host from the invasion of pathogens [74].

The epithelial cells are involved in immune defense mechanisms through the secretion of lysozyme, lactoferrin, IgM, and IgA [77], preserving a healthy nasal environment, and preventing infection and inflammation [78]. The nasal commensal bacteria inhibit the growth and colonization of the pathogens by releasing antagonistic chemicals, and through nutrition and space deprivation. On the other hand, dysbiosis can result in infections such as influenza [36]. The nasal microbial community reflects the health status and functionality, and can be used as an assessment tool for disease diagnosis [8,79].

The prime function of the human olfactory system is to discriminate between odors [80]. The olfactory function is facilitated by over 6,000,000 bipolar olfactory receptors, which are of central nervous system origin [81], and are present in the olfactory epithelium. The olfactory function is significant for human well-being and health; any dysfunction in olfaction can cause serious ailments, such as negative effects on mood, safety, the enjoyment of food, personal hygiene, social interactions, and sexual relations [82,83,84], and are associated with weight gain [85] and weight loss [86].

The microbiota are essential for normal olfactory epithelium development. Koskinen et al. studied the interrelationship between the nasal microbiome and the olfactory function. They revealed that the *Faecalibacterium* spp. and *Porphyromonas* spp. were involved in the decline in the olfactory function, and the *Corynebacterium* members were associated with a reduction in odor discrimination and threshold [78]. The high risk of olfactory dysfunction increases with age and other diseases, such as chronic diseases in the sinonasal regions (about the sinus and nasal regions), head trauma, URTIs, or neurological diseases. The microbiota of the lungs are likely to resemble that of the mouth. *Streptococcus*, *Prevotella* and *Veronica* spp. are most common in the lungs and the mouth [87]. Comparable to middle-aged adults, children and older adults are most susceptible to infections initiated from the URT, such as pneumonia [88] and influenza [89]. Towards the stage of middle-aged adults, the susceptibility rate decreases, with an increase in the immune responses of the mature URT [90,91]. Older adults are more susceptible to infections, due to the decline in the immune system [92] and the decrease in muconasal clearance [36].

The olfactory circuit was studied by exposing experimental subjects to specific olfactory tasks based on emotion, memory, and identification parameters. The results showed that an odor enters the olfactory system and travels to various brain regions, such as the entorhinal cortex, hippocampus, amygdala, orbitofrontal cortex, thalamus, and piriform cortex [93]. The piriform cortex and amygdala regions showed strong activation with odor stimuli and were found to be important for recognizing odor and strong emotional stimuli. All of the odors are initially encoded as objects in the piriform cortex [93]. Volunteers with hyposmia have reduced olfactory discrimination, and their nasal microbiota are rich in *Actinobacteria*, *Bacilli*, *Clostridia*, *Bacteroidia*, and *Proteobacteria*. *Corynebacterium* and *Faecalibacterium* are also involved in reduced odor discrimination. In addition, the nasal microbiota of subjects with a reduced odor threshold have an abundance of *Comamonadaceae* and *Enterobacteriaceae*. Butyrate-synthesizing *Porphyromonas* are associated with reduced olfactory function [94].

## 4. Olfactory Dysfunction and Neurological Disorders

Humans with olfactory impairment face many difficulties in their day-to-day lives [95]. Olfaction is one of the vital senses associated with human health and well-being. Olfactory dysfunction is an indicator of serious illnesses. The olfactory identification follows a different pathway, such as when the odor material binds to the olfactory receptors; the chemical binding elicits the electrochemical signals inside the olfactory neurons, the signals that are transmitted to the olfactory regions of the brain, later the autonomic nervous system and endocrine system are stimulated, which finally results in the emotional response [96]. Age is an important factor in olfactory dysfunction [97]. The olfactory ability declines mostly in middle-aged and older adults [98]. Schubert et al. [99] studied the risk of aging in olfactory dysfunction. Their results demonstrated that the risk of olfactory dysfunction was 4.1%, 21%, and 47.1% among 53–59 years, 70–79 years, and 80–97 years old, respectively. The results showed that the olfactory function deteriorates upon aging. Olfactory dysfunction is a primary indicator of the NDs [100]. A study about changes in olfaction during aging, and in certain neurological disorders, stated that olfaction is a complex sensory system known to affect cognitive abilities and mood. The neurophysiological features of the olfactory system and the odorant can lead to strong olfactory and emotional memories [101,102].

In addition to other external factors, the commensal organisms residing in the nasal cavity are also involved in developing the olfactory epithelium (OE). Evaluating the olfactory functions of the nasal microbiota proved that olfactory identification was not linked with the nasal microbiota. On the contrary, the olfactory threshold and olfactory discrimination are associated with the nasal microbial community [78]. Healthy volunteers were studied for their nasal microbiota and olfactory functions. The volunteers were categorized as per their olfactory ability, such as normal olfactory function, a good sense of smell, and hyposmia. The results indicated, surprisingly, that the microbial community in the nasal cavity differs between each group. The results showed that the nasal microbiota have a role to play in olfactory functions [78].

The microbes modulate the olfactory epithelium and influence the olfactory function [93]. The microbiome of the olfactory area is predominated by the phyla Actinobacteria, Firmicutes, Proteobacteria, and Bacteroidetes. Specifically, the species of *Corynebacterium*, *Staphylococcus*, and *Dolosigranulum* are abundant [68]. When the normal nasal microbiota get disturbed and are dominated by the commensal inhabitant, *Dolosigranulum pigrum*, olfactory infections occur [103]. The nasal microbiota and metabolites enter the brain through the olfactory system. The olfactory nerve from the nasal cavity enters the CNS bypassing the BBB, which results in the microbiota and its products accessing the olfactory bulb (OB) through the olfactory neuro epithelium [104]. The BBB is an interface for the blood–brain exchange [105], comprised of endothelial cells, astrocytes, neurons, and peripheral immune cells. Complex tight junctions regulate the movement of ions and macromolecules from systemic circulation at the inter-endothelial cleft. The microbial pathogens entry into the CNS is routed through the transcellular paracellular permeability [106].

**Figure 1:**
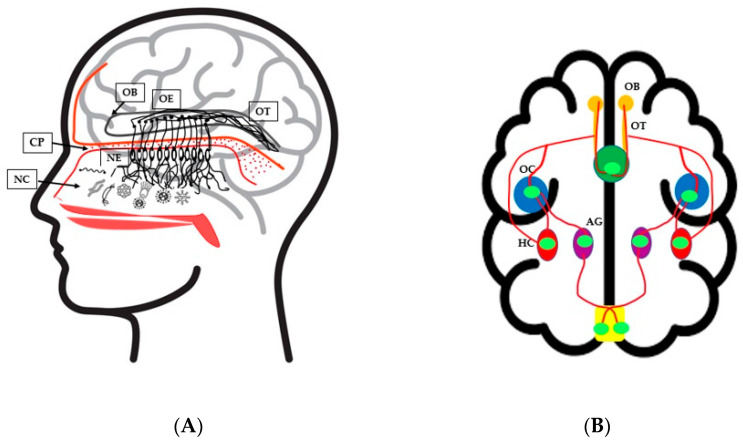
(**A**) Nasal microbiota interactions with the olfactory system that influence olfactory functions. Microorganisms enter through respiratory airways and interact with olfactory receptor cells (Orc) of the neuroepithelium (NE) that protrude through the perforations in the cribriform plate (CP) and extend its connections with olfactory neurons (ON) in the olfactory bulb (OB). The tuft of ONs forms the olfactory tract (OT), which connects to other olfactory cortices (OC); (**B**) Interactions of olfactory neurons within the brain. The tuft of olfactory nerve fibers from OT receives connections from the neurotransmitter system and OC. ON interacts with OC, hippocampus (HC), amygdala (AG), entorhinal cortex (EC), hypothalamus (HT), and locus coeruleus and reaches the reticular formation system (RFS), where the visceral responses of smell can be produced.

When microbes from aerosols or air enter the nasal cavity via the nostrils, it contacts the olfactory receptor cells (Orc). The axons of the Orc protrude into the neuroepithelium of the nasal cavity through the perforations in the cribriform plate (CP), and form synaptic connections with the neurons in the OB (Figure 1A) [104]. The tuft of olfactory nerve fibers receives connections from the neurotransmitter system and the olfactory cortices (OC). Olfactory signaling is a complex network where another set of olfactory neurons projects their nerve endings into the OC, hippocampus (HC), amygdala (AG), entorhinal cortex (EC), hypothalamus (HT), and locus coeruleus [106], and reaches the reticular formation system (RFS), which creates the visceral responses of smell (Figure 1B) [107]. Hence, the olfactory neuronal fibers extend their connection over the brain and enable the person to identify, discriminate, and correlate odors with emotions. The inflammatory cytokines and other immune regulators of the nasopharynx enter the brain’s extracellular fluid and the CNS [104] and can influence the olfactory function.

Olfactory dysfunction is common during aging, and, due to the structural changes in the nostrils, age-related alterations in the OE and OB [108]. Other reasons, such as chronic infections, age-related atrophy of the nasal epithelium, decreased mucosal blood flow, sympathetic and parasympathetic imbalance, nasal engorgement, abnormalities in the olfactory cortex (OC), sensory loss in the receptor cells, reduced mucosal enzymes, and changes in the neurotransmitter systems may induce olfactory impairment, which might cause cognitive and memory decline during aging and NDs, such as AD and PD [97]. Loss of integrity of the OE can occur during aging, due to a loss of the sensory responses in receptor cells, air-borne agents, smoking, and genetic factors [109]. Immunohistochemical studies revealed the presence of Aβ and paired helical tau elements in the OE of AD patients [110].

**Figure 2:**
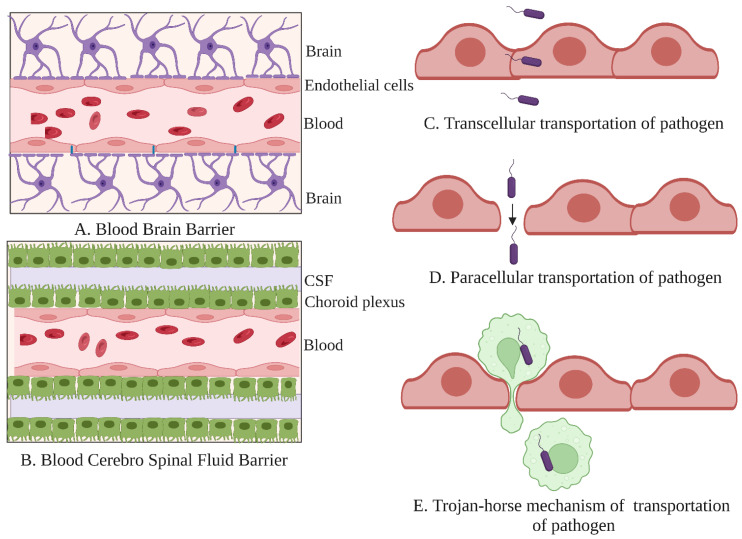
Barriers of the central nervous system. (**A**) The blood–brain barrier (BBB) is lined with tightly packed endothelial cells; (**B**) The blood–cerebrospinal fluid (CSF) barrier (BCSFB) is lined with a layer of endothelial cells and choroid plexus epithelial cells comprising CSF in between the choroid plexus epithelial cell layers. The possible means of bacterial entries, such as (**C**) transcellular transportation; (**D**) paracellular transportation; and (**E**) Trojan-horse mechanism. The illustration was created with BioRender.com.

Microbial pathogens can enter the CNS by penetrating the BBB, blood–cerebrospinal fluid barrier (BCSFB), and the olfactory and trigeminal nerves (Figure 2) [111]. The BCSFB is formed by endothelial and choroid plexus epithelial cells, producing CSF [112]. The pathogens cross the BBB either transcellularly or paracellularly or with the help of infected phagocytes using the Trojan-horse mechanism (Figure 2C–E). Thus, the pathogens disturb the BBB function, resulting in increased permeability, encephalopathy, or pleocytosis [113].

As a result of chronic inflammation and CRS, the homeostasis of the local microbiota gets altered, which could lead to the development of AD and dementia [114,115]. Hedner et al. analyzed the olfactory dysfunctions concerning cognitive demands using three parameters: odor threshold; odor discrimination; and odor identification using the Sniffin’ Sticks test [116]. The odor tests are effective as the odor representation is stored as a long-term memory. Hence, the brain can reveal and retrieve the odor later [117]. The smell helps perceive the external environment and other behaviors, such as decision-making, eating, detecting danger, etc. [117,118]. Anosmia, the complete loss of olfactory function, and hyposmia, decreased olfactory function, are common in neurological disorders [119,120]. Anosmia or hyposmia can result from various other reasons, such as head injury, cranial surgery, allergies, medication, cranial surgery, URTIs, and chemicals that cause nasal irritation. The available relevant investigations regarding olfactory and memory deficits showed that these are prevalent in NDs, such as AD, PD, MS, Huntington’s disease, and motor neuron disease [100].

The studies have examined the relationship between dementia and chronic rhinitis (CR), and CR is also related to other conditions, such as stroke, vasculopathy, and vascular dementia [121,122,123]. The patients with mild cognitive impairment and CR were more susceptible to the development of dementia than the patients without CR [124]. Chronic inflammation can be considered the key factor that bridges CR and AD. The dysregulated immune system in CR initiates inflammation. The pathology of CR involves a decrease in immunoglobulin J chain, antileukoproteinase, surfactant protein A [125], and an increase in the immune cells, eosinophils and basophils [126], which produce inflammatory cytokines IL-13, IL-5, IL-4 [127], IL-6, IL-12, IL-18, tumor necrosis factor-α (TNF-α), and transforming growth factor-β (TGF-β) in the mucosal region of CR patients. Thus, the increase in inflammatory cytokines, which disrupts the nasal epithelial cell regeneration by inhibiting the neural progenitor cell proliferation and provoking CR [128], might also lead to the disruption of neural integrity in the CNS and cause neurodegeneration [50]. The progression of CR pathology with aging causes damage to the olfactory neural epithelium [129,130].

Memory loss and cognitive impairments are the signature characteristics associated with AD. The main cause of dementia has been a global concern, due to the high risk of AD in the elderly [131]. Depression and cognitive impairment are the initial symptoms of AD, which consequently lead to severe memory loss, behavioral and personality changes, difficulties in executing day-to-day tasks, reduced communication capabilities [132], weakened immune function, and difficulties in movement and swallowing [133,134]. Currently, no direct evidence supports the association between AD pathology and the inflammatory responses of the nasal microbiota. However, in some cases, the cognitive dysfunction was improved through sinus therapies and CR treatments [135]. In addition to the OB infections, *C. pneumonia* was also found in the microglia, astrocytes, and neurons of patients who died of AD. Thus, the defensive functions of the astrocytes and microglia cells have deteriorated due to *C. pneumonia* invasion, which can consequently increase AD pathogenesis. *C. pneumonia* is a pneumonia-causing pathogen that has been one of the reasons for mortality in AD patients [136]. These studies support the involvement of respiratory pathogens in AD pathogenesis.

**Figure 3:**
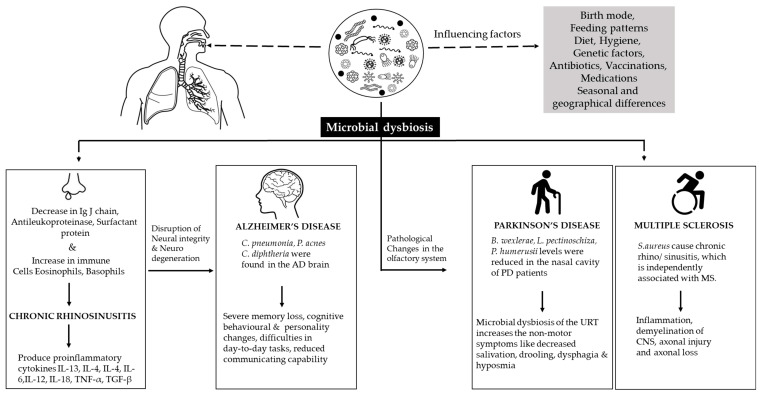
Illustration representing the correlation between nasal microbial dysbiosis and chronic rhinosinusitis (CRS), Alzheimer’s disease (AD), Parkinson’s disease (PD), and multiple sclerosis (MS). *C. pneumonia* (*Chlamydia pneumoniae*); *P. acnes* (*Propionibacterium acnes*); *C. diphtheria* (*Corynebacterium diphtheriae*); *B. wexlerae* (*Blautia wexlerae*); *L. pectinoschiza* (*Lachnospira pectinoschiza*); *P. humerusii* (*Propionibacterium humerusii*); *S. aureus* (*Staphylococcus aureus*).

Olfactory dysfunction is one of the biomarkers of physiological decline [137], heart failure, stroke, diabetes, hypertension, liver damage, and even cancer [100], and is also predicted as a high-risk marker of psychosis [138] and other neurological diseases, such as MS and epilepsy [84]. The oral and nasal regions are two important entry points for pathogens; they later spread to the CNS and lead to PD pathogenesis [139]. The link between PD and nasal microbiota has been examined. The results indicated that the dysbiosis of the nasopharyngeal microbiota creates the inflammatory response to α-synuclein that ends in neurodegenerative disorders [140,141]. The accumulation and aggregation of α-synuclein in the dopaminergic substantia nigra of the CNS cause a neuronal loss in PD [142]. Most PD patients showed olfactory deficits in the early period of the disease before the occurrence of the motor symptoms [143]. Clinically, PD patients experience non-motor symptoms (NMS), such as decreased salivation, drooling, dysphagia, and hyposmia. These NMS are related to the pathological changes in the olfactory system [144]. These studies suggested the involvement of the nasal microbial community in the progression of PD. In contrast, the relative abundance of non-inflammatory bacteria, such as *Blautia wexlerae*, *Lachnospira pectinoschiza*, and *Propionibacterium humerusii*, were reduced in the nasal sinus cavity of PD patients (Figure 3) [145].

## 5. Diet–Microbiota–Brain Interrelationship

Over the past few decades, a greater priority has been given to studying the gut microbiota–diet–brain interrelationship. The changes in the neurochemical profile and abnormal behaviors recorded in the animal model showed that the gut microbiota are involved in the brain development and functions [146,147]. The gut microbiota composition of an individual could modulate the diet-dependent gut microbiota metabolites [148].

The gastrointestinal (GI) tract wall is innervated with the CNS through the enteric nervous system (ENS) [149]. It is predominantly colonized by Bacteroidetes and Firmicutes [150], Actinobacteria, Cyanobacteria, Fusobacteria, Proteobacteria, and Verrucobacteria [151], which can directly or indirectly influence the host immune responses [152]. Firmicutes comprise genera, such as *Clostridium*, *Bacillus*, *Lactobacillus*, *Enterococcus*, and *Ruminococcus*. The Bacteroidetes consist of genera *Bacteroides* and *Prevotella* [67]. In the same way as the gut, the phyla Actinobacteria, Bacteroidetes, Firmicutes, and Proteobacteria are members of healthy nasal microbiota [16,67,72,153]. The adequate moist environment in the nasal passage has been termed as an access point for microbes from the environment. Proteobacteria and Actinobacteria are the most predominant in the brain; any disruption may cause CNS disorders [154].

MS is a demyelinating inflammatory disorder linked with CNS dysbiosis. The studies in the frozen and autopsied brain samples of MS patients revealed the presence of the dominant phylum, Proteobacteria [155]. AD brains showed an increased number of Actinobacteria compared to the controls [22]. The proofs exist for the gut and CNS dysbiosis and their impact on neurodegeneration. However, the in vivo studies on the dysbiosis of other mucosal surfaces, such as the nasal region, related to neurological disorders is limited [104]. However, the direct relationship between mucosal dysbiosis and neurodegenerative disease processes, and the data on how the microbiota at these mucosal surfaces trigger CNS inflammation and neurodegeneration, require further investigation [104]. The axons of the ORc enter the cribriform plate and form synapses with the OBs neurons. As the lymphatics of the posterior paranasal sinuses drain into the extracellular fluid of the brain, the by-products of the nasal microbiota can access the CNS [104]. Gut dysbiosis causes the accumulation of lipopolysaccharides (LPS), pro-inflammatory cytokines, Helper T cells and monocytes, which in turn lead to the loss of integrity of the intestine and the BBB, which subsequently results in the pro-inflammatory conditions, and the pathogenesis of NDs [156]. A diet rich in highly processed carbohydrates can produce high levels of inflammatory cytokines [157]. On the contrary, a diet rich in fibers can help reduce gut and systemic inflammation, lower the inflammatory cytokines, and enhance the synthesis of SCFAs, such as butyrate, acetate, and propionate, which help maintain gut microbiota diversity [158].

Bacteroidetes and Firmicutes produce acetate and propionate, and Firmicutes produce butyrate. These metabolites maintain gut homeostasis and anti-inflammatory actions. The microbial metabolites depend on the diet and baseline microbiota composition [159,160]. They also act as a driving force for intestinal epithelial cells and enhance the gut barrier functions [34]. Butyrate acts as the main energy source for colonocytes [161]. The gut microbes, *Faecalibacterium prausnitzii*, *Clostridium leptum*, *Eubacterium rectale* and *Roseburia* spp., are known to produce butyrate [162,163]. The lactate-dependent bacteria, *Eubacterium hallii*, and *Anaerostipes caccae*, synthesize both butyrate and acetate [164].

SCFAs, such as propionate, inhibit interleukin-17 (IL-17) production in mice and humans [165]. The interplay between the microbiota in the mucosal surfaces, such as the gut, nasal cavity, and immune cells, is complex and organized as a circuit, where the signals transmit between the microbes–nervous system–immune system. This circuit undoubtedly depends upon the dietary composition ingested by the individuals. Hence, the interrelationship among the diet–gut–nasal microbiota–brain is considerable in the case of immune and neuro functions and pathogenesis. The diet is a key to maintaining a safe and healthy life in coordination with the gut microbiome. Even though studies about the diet–microbiome–mucus interactions are budding here and there, the understanding of the linear interactions of diet–gut microbiome–mucus with inflammatory infections and neurological diseases needs more clinical evaluation [30].

Healthy diets could restore the beneficial microbiota and promote health. The diet predominantly influences the gut microbiota and strongly correlates with food-associated health issues [166]. The influence of the diet on the immune system during COVID-19 was reported [167]. COVID-19 patients could lose their nutritional status due to anorexia, vomiting, nausea, diarrhea, hypermetabolism, and nitrogen loss. The dietary components may interact with the ACE-2 receptor, the hallmark entry point of SARS-CoV-2, and reduce the inflammatory response caused by SARS-CoV-2 [167]. Respiratory infections, such as COVID-19, can stimulate the synthesis of inflammatory cytokines in the host system. To balance the host immune system, dietary practices, such as limiting the carbohydrate-rich diet and a including a fiber-containing diet, can be initiated in the case of COVID-19 infection, to control the synthesis and circulation of the inflammatory cytokines [167].

## 6. Nasal Microbiota and COVID-19

COVID-19 is an RTI, resulting in high mortality and morbidity worldwide, and the survivors are at a high risk of developing neurological disorders [168]. Some studies evaluated that the SARS-CoV-2 virus may cross the BBB and OB and enter the CNS [169,170]. The human URT is the major entry route for SARS-CoV-2 [171] and is transmitted through sneezing, coughing, or speaking with an infected person at a close distance. COVID-19 may evoke severe neurodegeneration, which could reduce the overall survival of COVID-19 patients with AD [172]. Few studies report the association between CoVs with CNS diseases, such as MS, acute disseminated encephalomyelitis [173,174,175], febrile seizures, and encephalitis epilepsy [176]. SARS-CoV-2 can enter the host through the upper airways. The viral replication occurs in the upper respiratory epithelia and is transmitted through ACE-2, resulting in immune responses [177]. SARS-CoV-2 infection results in a systemic increase in the pro-inflammatory cytokines, virtually resulting in neuroinflammation [178]. The SARS-CoV-2 enters the CNS by binding to the ACE-2 receptor in the endothelial cells of the BBB, and bypassing the BBB protection by targeting the CNS macrophages and monocytes [179].

De Maio et al. reported that the microbiota in the nasopharynx were not altered in COVID-19 patients, especially the phyla Firmicutes, Bacteroidetes, Proteobacteria, Actinobacteria, and Fusobacteria which were found in both SARS-CoV-2 infected and uninfected subjects [180]. The members of the phyla Firmicutes, Bacteroidetes, Proteobacteria and Actinobacteria, and genera *Streptococcus*, *Prevotella*, *Veillonella*, *Haemophilus*, and *Moraxella* were abundantly present in the nasopharynxes of COVID-19 patients [181].

Usually, the genus *Prevotella* is considered commensal, but some strains rarely cause infections [182]. In the COVID-19 pathological condition, *Prevotella* induces the production of cytokines and inflammation through T helper 17 cells [183]. The *Prevotella* proteins promote viral infection and COVID-19 severity through NF-κB signaling [184]. A good balance in the URT is maintained with the help of healthy nasal microbiota. In the case of COVID-19, the gut and LRT microbiota are altered because of therapeutic interventions [185,186,187].

The COVID-19 infection is associated with neurological manifestations. Nowadays, studies are emerging to decode the role of SARS-CoV-2 in CNS pathophysiology. Experimental evidence showed that the human CoVs could infect the neurons, microglia, astrocytes, and glial cells and confirmed the presence of CoVs in the autopsies of MS, PD, and AD patients [188]. A study reported that about 20% of the COVID-19 patients admitted to intensive care units (ICU) showed neurological complications, including stroke, encephalopathy, acute inflammatory demyelinating polyneuropathy, and encephalitis [189]. Strokes commonly occur as a COVID-19 complication in a few older, hypertensive, and hemorrhagic cases [190]. Dementia patients are at an increased risk of COVID-19 infection [191]. PD patients are at a higher risk of COVID-19-associated mortality than non-PD patients [192]; they also showed worsening PD symptoms, such as rigidity, fatigue, tremor, and pain [193], and an increase in depression, insomnia, and irritability [193,194]. Other neurological conditions, such as confusion, unconsciousness, agitation, seizure, and altered mental states, were observed in COVID-19 patients [195,196]. Neuropsychiatric complications, such as symptoms of post-traumatic stress disorder, depression, anxiety, obsessive-compulsive disorder, and insomnia were also reported in the hospitalized COVID-19 patients [197].

## 7. Conclusions

Microbes survive within the host either as commensals or as opportunistic pathogens. The mucosal membranes provide shelter for the microbes, and the microbiota are involved in various functions, such as metabolism, immune responses, and pathogen resistance. Maintaining healthy mucosal environments is essential to sustaining healthy microbiota. The nasal microbiota are potentially connected with the olfactory function by modulating the physiology of the olfactory epithelium. The incidence of olfactory dysfunction increases during aging, due to the loss of olfactory NE and the reduced activity of the olfactory cortex. Respiratory infections may cause olfactory dysfunction. The invasive pathogens enter the nasal cavity and bind to the olfactory receptors of the olfactory NE. They could result in inflammatory changes, olfactory impairment, and even temporary or permanent olfactory dysfunction.

Diet plays an indomitable role in shaping the human microbiota and maintaining interactions between the microbiota–host–mucosal environments. Dietary changes affect the mucosal barriers with or without the involvement of the microbiota. The diet’s macro- and micronutrients, lipids, proteins, and carbohydrates may differentially regulate the prevalence of microbiota. A diet rich in fiber can limit the production of inflammatory cytokines by the gut microbiota. Hence, maintaining a healthy microenvironment for gut functioning during respiratory infection is necessary. The increase in respiratory, inflammatory, and neurological diseases has been a more alarming health threat globally. The involvement of nasal–gut microbiota in inflammatory diseases is explained through various studies, but the etiology of neurological diseases is not clearly stated. Thus, more studies are necessary to unveil the correlation between the nasal microbiota and the nervous system.

## Figures and Tables

**Table 1:** Representing different microbial communities residing in the respiratory tract across the ages.

S. No.	Samples	Experimental Subjects	Study Methodology	Commensal Microbiota	Reference
1	Anterior nares and Oropharynx	Elderly participants (age 68 to 96 years)	16S rRNA gene sequencing	*Propionibacterium* spp., *Corynebacterium* spp., *Staphylococcus* spp., *Veillonella* spp., *Streptococcus* spp.	[16]
2	Nasal and Oropharynx	Elderly participants (age ≥ 65 years); 152 controls and 152 patients with RTIs.	16S rRNA gene sequencing, quantitative real-time PCR, and culture.	In the nasal passage: *Corynebacterium*, *Staphylococcus*, *Moraxella*, *Dolosigranulum*, *Streptococcus*, *Haemophilus*, *Peptoniphilus*, *Cutibacterium*, *Anaerococcus*, and *Enterobacteriaceae*. Less abundantly: *Pseudomonas* and *Neisseria*. In the oropharynx: *Prevotella*, *Veillonella*, *Streptococcus*, *Neisseria*, *Fusobacterium*, *Leptotrichia*, *Haemophilus*, *Rothia*, *Porphyromonas*, *Actinobacillus*, *Lactobacillus*, *Staphylococcus*.	[19]
3	URT, Nasopharynx	Healthy children (*n* = 60); Age 1.5, 6, 12, and 24 months.	16S rRNA-based pyrosequencing	At 1.5 months of age: *Staphylococcus* sp., *Corynebacterium* sp., *Moraxella* sp. At 1.5 to 6 months of age: *M. catarrhalis*, *Dolosigranulum* sp., *Corynebacterium* sp. At 6 months of age: *Staphylococcus aureus* In the first 2 years of age: *Moraxella* sp., *Dolosigranulum* sp., *Corynebacterium* sp., *Haemophilus* sp., *Streptococcus* sp.	[20]
4	URT, Nasopharynx	Infants exclusively breastfed (*n* = 101) and exclusively fed formula (*n* = 101); Age 6 weeks to 6 months.	16S-based GS-FLX-titanium-pyrosequencing	Breastfed infants: Abundance of *Dolosigranulum* sp., *Corynebacterium*, *pseudodiphteriticum*, *C. propinquum*, *C. accolens*, *C. fastidiosum*, or *C. segmentosum*, Decreased abundance of *Staphylococcus* spp., *Prevotella* sp., *Veilonella* sp. Formula-fed infants: *Dolosigranulum* sp., *Corynebacterium* sp.	[39]
5	URT, Nasopharynx	A cohort of 234 children, including healthy infants and infants who had experienced acute respiratory infections once.	Microbial profiling using 16S rRNA gene deep sequencing	*Staphylococcus* sp., *Corynebacterium* sp., *Alloiococcus* sp., *Moraxella* sp., *Haemophilus* sp.	[41]
6	URT-anterior nares (left and right)	Healthy adults and hospitalized patients. *S. aureus* carriers (*n* = 26) and non-carriers (*n* = 16).	Culture-independent analysis of 16S rRNA sequencing	Actinobacteria (*Propionibacterium* sp., *Corynebacterium* sp.) Firmicutes (*Staphylococcus* spp.), Proteobacteria (*Enterobacter* sp.)	[58]
7	URT-anterior nares	A healthy cohort of 236 subjects from the Human Microbiome Project.	16S rRNA gene sequencing	*Moraxella* sp., *Corynebacterium* sp., *Propionibacterium* sp., *Staphylococcus* sp.	[59]
8	URT-anterior and posterior vestibule, inferior and middle meatuses of the nasal passage	A cohort of CR and CR-free individuals (*n* = 79).	Illumina paired-end sequencing of the V1-V2 variable regions of the 16S rRNA gene.	*Staphylococcusaureus*, *Moraxella* sp., *Finegoldia* *magna*, *Prevotella* sp., *Staphylococcus epidermidis*, *Haemophilus influenzae*.	[60]
9	URT, Nasopharynx	The unselected birth cohort of healthy children born by Cesarean (*n* = 40) and vaginal birth (*n* = 62); Age (birth to 6 months).	Constructing the phylogenetic library by amplifying hypervariable v4 region of 6s rRNA gene	*Staphylococcus aureus*, *Streptococcus viridans*, *S. pneumoniae*, *Corynebacterium pseudodiphteriticum*, *C. propinquum*, *Dolosigranulum pigrum*, *Moraxella catarrhalis*, *M. nonliquefaciens*, *Haemophilus influenzae*	[61]
10	The right and left nasopharynx and oropharynx	Smoking (*n* = 29) and non-smoking (*n* = 33) healthy asymptomatic adults	16S rRNA-based pyrosequencing	The nasopharynx is dominated by *Firmicutes*, *Proteobacteria*, *Bacteroidetes*, *Actinobacteria*, and *Campylobacter* sp. *Streptococcus*, *Shigella*, *Acinetobacter*, and *Corynebacterium* sp. The oropharynx is dominated by *Streptococcus Bacteroidetes*, *Firmicutes*, *Proteobacteria*, and *Fusobacteria*. *Prevotella*, *Fusobacterium*, *Neisseria*, *Leptotrichia*, and *Veillonella* sp.	[62]
11	Nasal cavity and dust samples	A cohort of healthy volunteers (*n* = 50) without a history of respiratory system diseases.	Standard mycological techniques based on gross cultural and microscopic morphology	*Aspergillus*, *Penicillium*, *Yeast*, *Alternaria* and *Rhizopus*	[63]
12	Nasopharynx	Children (Age < 6 years; *n* = 135) with and without severe acute respiratory infections (SARI)	Metagenomic analysis based on Next-Generation Sequencing	In children with SARI: Members of the *Paramyxoviridae*, *Coronaviridae*, *Parvoviridae*, *Orthomyxoviridae*, *Picornaviridae*, *Anelloviridae* and *Adenoviridae* In children without SARI: Members of *Anelloviridae*	[64]
13	Oropharynx Nasopharynx Bronchoalveolar	Children with or without lung infection (*n* = 78)	16S rRNA gene sequencing	*Moraxella*, *Haemophilus*, *Staphylococcus*, *Streptococcus*, *Neisseria*, *Prevotella* and *Corynebacterium* spp.	[65]
14	Bronchoalveolar	Asymptomatic subjects. Never-smokers (*n* = 9), former-smokers (*n* = 14), and current-smokers (*n* = 6).	16S rRNA gene sequencing	*Propionibacterium*, *Staphylococcus*, *Corynebacterium*, *Stenotrophomonas*, *Pseudomonas*, *Prevotella*, *Veillonella*, *Streptococcus*, *Fusobacterium*, *Porphyromonas*, *Sphingomonas*, *Tropheryma*, *Acidovorax*, and *Asticcacaulis*	[66]
15	oropharynx and nasopharynx	Healthy children (Age 1 to 4.5 years; *n* =51) and accompanying parents (*n* = 19).	Molecular profiling of the bacterial 16S rRNA gene	Oropharynx of both children and adults: *Streptococcus* sp., *Rothia* sp., *Prevotella* sp. *Gemella* sp., *Veillonella* sp., *Fusobacteria* sp., *Haemophilus* spp., *Neisseria* sp. Nasopharynx of adults: *Firmicutes* sp., *Staphylococcus* sp., *Streptococcus* sp., *Bacteriodetes* sp., *Prevotella* sp., *Actinobacteria* sp., *Corynebacterium* sp., *Rothia* sp., and *Propionibacterium* sp., Nasopharynx of children: *Moraxella* spp., *Enterobacteriaceae* sp., *Haemophilus* sp., *Enterococcus* sp.	[67]

## Data Availability

The data presented in this study are available within the article.
